# Damage Identification Method of Box Girder Bridges Based on Distributed Long-Gauge Strain Influence Line under Moving Load

**DOI:** 10.3390/s21030915

**Published:** 2021-01-29

**Authors:** Jing Yang, Peng Hou, Caiqian Yang, Ning Yang, Kefeng Li

**Affiliations:** 1 Key Laboratory of Concrete and Prestressed Concrete Structures of the Ministry of Education, School of Civil Engineering, Southeast University, Nanjing 210096, China; yangjingseu@seu.edu.cn (J.Y.); houpeng@seu.edu.cn (P.H.); likefengseu@seu.edu.cn (K.L.); 2 College of Civil Engineering & Mechanics, Xiangtan University, Xiangtan 411105, China; 3 Shandong Institute of Space Electronic Technology, Yantai 264670, China; yn3714@163.com

**Keywords:** damage identification, moving load, long-gauge fiber Bragg grating strain sensor, influence line, damage extent

## Abstract

A new method was proposed for the damage identification of box girder bridges under moving load, wherein the difference of strain influence line (DSIL) was taken as an index to represent the long-gauge strain difference before and after damage. The damage identification theory based on long-gauge strain influence lines was derived for box girder bridges with shear lag effect under consideration, and a regularized index DSIL was proposed for the quantitative identifications of damage location and extent. A series of experiments were carried out to study the influences of speed, vehicle type, and vehicle weight on the damage identification, and the experimental data were obtained by long-gauge fiber Bragg grating strain sensors. Moreover, numerical simulations were performed to confirm the method. The experimental and numerical results show that the method can locate the damage accurately, and quantitatively identify the damage extent under different working conditions. The experimental damage extent is generally slightly higher than the theoretical, with an average identification error smaller than 5%. Additionally, the relative error of damage extent is smaller than 3% under different working conditions. Thus, the effectiveness of this method was verified.

## 1. Introduction

As important transportation infrastructure, bridges occupy an important position in national economic development. Concrete box girder bridges are widely used on medium- and long-span bridges due to their unique cross-sectional form and usage characteristics. However, they are affected by traffic load, overload, environmental erosion, earthquakes, and typhoons in daily operation, which will inevitably lead to structural damage. All these result in decreases in structural stiffness, bearing capacity, and service life span, and even in abrupt collapse. Therefore, it is of great significance to study and develop a structural damage identification and warning method suitable for box girder bridges in order to detect their early structural damage and evaluate the service state and safety of the box girder bridges, which is in favor of a reasonable maintenance and management.

Bridge structural health monitoring (SHM) has attracted the extensive attention of many researchers [1,2,3,4,5] and has been successively applied to bridge structures as an important part of disaster reduction. As a core issue in SHM, structural damage identification technology has developed rapidly. Damage identification technology can be grossly divided into two species: frequency-domain signal (FDS)-based methods, and time-domain signal (TDS)-based methods [6]. FDS-based methods mainly use the dynamic characteristics of the structure, such as natural frequency, mode, etc., to identify the damage and parameters of the structure, and then to evaluate its working state and practical life; extensive literature reviews on FDS-based methods have been conducted [7,8,9,10,11]. However, this method is not sensitive to local structural damage in practical application, which leads to the deviation of identification. Some FDS-based methods can avoid this problem and are more sensitive to local damage. However, it is greatly prone to external load, which obstruct its development [12,13,14].

Vibration-based damage identification (VBDI) techniques developed rapidly in the 2010s. The core of the technology is that the vibration characteristics of the structure are a function of its physical parameters. If the structure is damaged, it means a change in the structural parameters, so it will inevitably lead to the change in the structural vibration characteristics. Doebling et al. [15] summarized the structural damage identification methods based on the dynamic characteristic change of a structural system in detail; its weakness is that the structural overall parameter is used to identify the local damage. Lee et al. [16] presented a bi-level damage detection algorithm that utilizes dynamic responses of the structure as the input and a neural network as a pattern classifier, but it is difficult to use in practical engineering. Ntotsios et al. [17] proposed to extract damage indicators from acceleration signals, but this type of method is too affected by noise, and the obtained structural information is too macro to capture the local damage. Li et al. [18] presented a distributed damage identification approach based on the dynamic response sensitivity of a moving vehicle. Liu et al. [19] used the data from strain gauges installed on bridges and finite element simulation to generate three types of sensor networks, and the causal relationships among spatially distributed strain data streams were extracted and analyzed for the localization of structural degradation in bridges. Nguyen et al. [20] presented a novel damage identification method to locate and quantify damage using measured mode shapes and natural frequencies.

The method of identifying damage by strain response has developed rapidly. However, the traditional strain measurement is basically “point” measurement, in which it is difficult to effectively capture the structural damage. In order to solve this problem, Li and Wu [21] proposed the concept of a distributed long gauge strain sensing, and developed the related techniques and sensors. Liu et al. [22] applied the Brillouin optical time domain analysis (BOTDA) technique to bridge damage localization and proposed a damage localization index based on quasi-static strain influence lines which was independent of differences in the loading conditions before and after damage. Sun et al. [23] proposed a novel real-time damage identification method for bridge SHM considering temperature variation; the method utilized model-based and partial least-squares regression analyses for damage identification. Zhao et al. [24] proposed a damage identification technique based on distributed strain measurements, identifying the structural damage under ambient excitation; the distributed strain energy difference and the relative distributed strain energy were used to identified and quantify damage. Hong et al. [25,26] proposed a damage-assessment method based on long-gauge strain sensors, which is used for the rapid diagnosis and long-term health monitoring of structures. However, the method needs a reference element. Chen et al. [27] carried out a strict comparative study on three representative methods using long-gauge fiber Bragg grating (FBG) for damage detection of highway bridges, and the precision and reliability of three methods were thoroughly studied and compared through the vehicle bridge coupling experiment. Wu et al. [28] proposed a novel method for stiffness monitoring and damage identification of bridges under moving vehicle loads using spatially-distributed optical fiber sensors. However, there are few damage identification methods for the box girder bridges mainly applied in actual engineering.

In this article, a damage identification method of box girder bridges is proposed, based on long gauge strain influence lines. The feasibility and identification effects of this method were verified by numerical simulations and model bridge experiments, and the influence of vehicle speed, vehicle type, axle loads, and other parameters were studied on the damage identification of box girder bridges. The results show that the method can be used to locate the damage of the box girder bridges and quantify the extent of the damage. 

## 2. Damage Identification of Box Girder Bridges Based on Strain Influence Lines

The damage identification method of box girder structures under moving load is studied by using strain influence line theory [29] and distributed long-gauge strain sensing technology. Firstly, the equation of long gauge strain influence lines of box girder bridges is derived.

**Figure 1:**
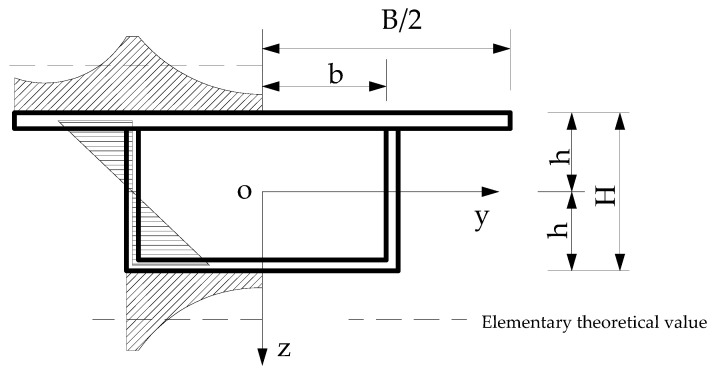
Transverse distribution of bending normal stress in rectangular box girder.

For the thin-walled rectangular box section shown in Figure 1, the expression of bending normal stress of wing plate considering the effect of shear lag [30] is:

(1) $$\sigma_{x}=Eh\left[\frac{M(x)}{EI}-(1-{\bar{y}}^{3}-\frac{3I_{s}}{4I})u^{\prime }\right]$$

in which:

(2) $$u(x)=\frac{7n}{6EI}\left(c_{1}shkx+c_{2}chkx+u^{*}\right)$$

(3) $$\left\{ \begin{array}{l} n=\frac{1}{1-\frac{7}{8}\frac{I_{s}}{I}}\\ \;k=\frac{1}{b}\sqrt{\frac{14Gn}{5E}} \end{array}\right.$$

where *u*(*x*) is the shear displacement function of the wing plate of the box girder, *c*_1_ and *c*_2_ are determined by the boundary conditions, u* is the special solution only related to the shear distribution, *h* is the height of the neutral axis, *M*(*x*) is the bending moment, *I_s_* is the inertia moment of the wing plate, *I* is the total inertia moment of the box girder, *EI* is the section stiffness, and *G* is the shear modulus, $\bar{y}=\frac{y}{b}$.

**Figure 2:**
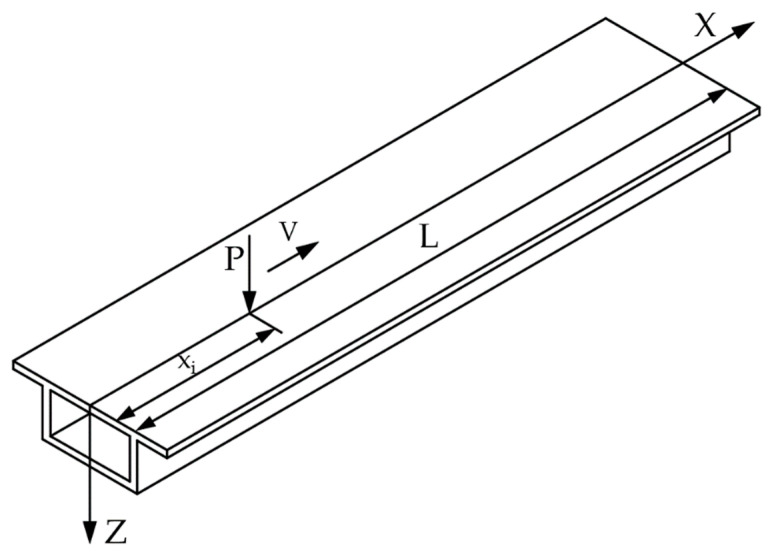
Simply supported beam structure under moving load.

For the simply supported box girder with uniform section shown in Figure 2, suppose a moving load *P* acting symmetrically on the rib plate of the box girder, according to the basic assumption of the variational method, then:

(4) $$\left\{ \begin{array}{l} M_{1}(x)=\xi Px\\ u_{1}=\frac{7nP}{6EI}(c_{1}shkx+c_{2}chkx+\frac{\xi }{k^{2}}) \end{array}\right.\;0\leq x\leq x_{i}$$

(5) $$\left\{ \begin{array}{l} M_{2}(x)=(x_{i}-\eta x)x\\ u_{2}=\frac{7nP}{6EI}(c_{3}shkx+c_{4}chkx+\frac{\eta }{k^{2}}) \end{array}\right.\;x_{i}<x\leq L$$

where $\xi =\frac{L-x_{i}}{L}$, $\eta =\frac{x_{i}}{L}$ is the boundary condition; ${u^{\prime }}_{1|x=0}=0$, ${u^{\prime }}_{2|x=l}=0$; continuous condition: $x=x_{i}$, $u_{1}=u_{2}$, from the variational condition, at point $x=x_{i}$:

(6) $$\left({u^{\prime }}_{1}-\frac{7nM}{6EI}\right){|}^{x_{i}}+\left({u^{\prime }}_{2}-\frac{7nM}{6EI}\right){|}_{x_{i}}=0$$

According to the above boundary conditions:

(7) $$\left\{ \begin{array}{l} u_{1}=\frac{7nP}{6EIk^{2}}(\frac{shk(L-x_{i})}{shkL}chkx-\zeta )\\ u_{2}=\frac{7nP}{6EIk^{2}}(shkx_{i}shkx-shkx_{i}cthkLchkx+\eta ) \end{array}\right.$$

By introducing Equations (2)–(7) into Equation (1), the normal bending stress of the structure can be obtained as:

(8) $$\sigma_{x}=\left\{ \begin{array}{l} \frac{h}{I}\left\{M(x)-\frac{7nP}{6k}(1-\frac{3I_{s}}{4I})\left[\frac{shk(L-x_{i})}{shkL}shkx\right]\right\}\;(0\leq x_{i}\leq x)\\ \frac{h}{I}\left\{M(x)-\frac{7nP}{6k}(1-\frac{3I_{s}}{4I})\left(shkx_{i}\cdot chkx-shkx_{i}\cdot cthkL\cdot \;shkx\right)\right\}\;(x<x\leq L)\; \end{array}\right.$$

According to structural mechanics, the strain influence line equation at any section *x_i_* of a simply supported beam can be expressed as:

(9) $$f(x_{i})=\frac{\sigma_{x}}{{\left(E\right)}_{i}}$$

**Figure 3:**
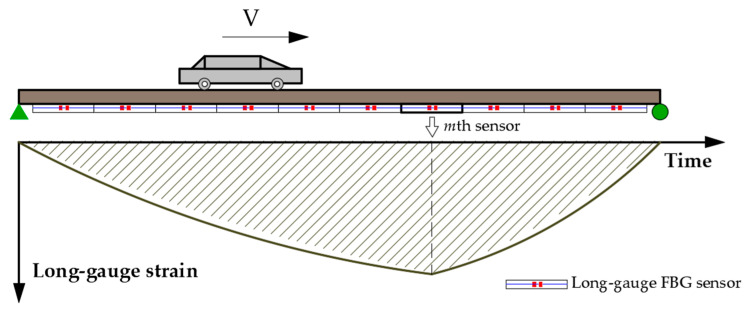
Schematic diagram of moving load passing over the bridge.

As shown in Figure 3, a moving load passes through the bridge at a uniform speed. Suppose that the bottom of the beam is divided into *N* units average along the length, and a long gauge FBG sensor is installed under each unit. Combining Equations (8) and (9), the expression of the long gauge strain influence line measured by the *m*th sensor at the bottom of the beam is as follows:

(10) $$f_{m}(x)=\left\{ \begin{array}{ll} \frac{h}{{\left(\bar{EI}\right)}_{m}}\left[\frac{\left(L-x_{i}\right)}{L}x-\lambda_{1}\cdot shkx\right] & \;(0\leq x\leq x_{i},1\leq m\leq N)\\ \frac{h}{{\left(\bar{EI}\right)}_{m}}\left[x_{i}(1-\frac{x}{L})-\lambda_{2}\cdot (chkx-cthkL\cdot shkx)\right] & \left(x_{i}<x\leq L,1\leq m\leq N\right) \end{array}\right.$$

where $\lambda_{1}=\frac{7nP}{6k}(1-\frac{3I_{s}}{4I})\frac{shk(L-x_{i})}{shkL}$, $\lambda_{2}=\frac{7nP}{6k}(1-\frac{3I_{s}}{4I})shkx_{i}$, and ${\left(\bar{EI}\right)}_{m}$ represent the equivalent stiffness of the structure within the gauge length of the *m*th sensor. Suppose that damage occurs in the *m*th element, the stiffness of the damaged element is ${\left(\bar{EI}\right)}^{*}{}_{m}=(1-\alpha ){\left(\bar{EI}\right)}_{m}$, *α* is the index of stiffness degradation degree, then the long gauge strain influence line of the *m*th sensor in the damaged state is as follows:

(11) $$f_{m}{\left(x\right)}^{*}=\frac{h}{{\left(\bar{EI}\right)}^{*}{}_{m}}\left[\frac{\left(L-x_{i}\right)}{L}x-\lambda_{1}\cdot shkx\right]\;\;(0\leq x\leq x_{i},1\leq j\leq N)$$

Calculating the difference between Equations (10) and (11), it can be obtained that:

(12) $$\begin{array}{l} \Delta f_{m}(x)=f_{m}{\left(x\right)}^{*}-f_{m}(x)=\left[\frac{h}{{\left(\bar{EI}\right)}^{*}{}_{m}}-\frac{h}{{\left(\bar{EI}\right)}_{m}}\right]\left[\frac{\left(L-x_{i}\right)}{L}x-\lambda_{1}\cdot shkx\right]\\ =\frac{\alpha }{1-\alpha }\frac{h}{{\left(\bar{EI}\right)}_{m}}\left[\frac{\left(L-x_{i}\right)}{L}x-\lambda_{1}\cdot shkx\right]\\ =\frac{\alpha }{1-\alpha }f_{m}(x) \end{array}$$

Equation (12) shows that the strain difference measured by the *m*th sensor before and after the damage is inversely proportional to the average stiffness in the sensor gauge length. The initial stiffness of the structure is a constant value, and the parameter can be used as a damage index to identify the structure damage based on this relationship. In order to facilitate comparison, a regularized damage index difference of strain influence line (DSIL) is proposed for the bridge damage identification. The expression of the damage indicator *DSIL* is:

(13) $$\begin{array}{ll} DSIL & =\left[DSIL_{1},DSIL_{2}\ldots DSIL_{m}\ldots DSIL_{N}\right]\\ & =\left\{\frac{{\left[\Delta f_{1}(x)\right]}_{\max}}{{\left[f_{1}(x)\right]}_{\max}},\frac{{\left[\Delta f_{2}(x)\right]}_{\max}}{{\left[f_{2}(x)\right]}_{\max}}\ldots \frac{{\left[\Delta f_{m}(x)\right]}_{\max}}{{\left[f_{m}(x)\right]}_{\max}}\ldots \frac{{\left[\Delta f_{N}(x)\right]}_{\max}}{{\left[f_{N}(x)\right]}_{\max}}\right\} \end{array}$$

where ${\left[f_{m}(x)\right]}_{\max}$ is the maximum amplitude of the strain influence line measured by the *m*th sensor before the damage. After regularization, the damage index DSIL is only related to the stiffness of the bridge structure and the position of the sensor, and independent of the load. Therefore, DSIL can be used to identify damage and evaluate structural performance. When the bridge is in a non-destructive state, the damage index DSIL is always around zero. However, when the structure within a certain sensor gauge length is damaged, the structural stiffness will decrease, and the corresponding value of DSIL will deviate from zero. This result can be used to the locate the damage of the structure.

For the quantification of structural damage degree, suppose that the structure is damaged within the *m*th sensor gauge distance, and *α* represents the damage degree. According to the definition of damage index DSIL, the expression of damage degree *α* is:

(14) $$\alpha =\frac{{\left(DSIL\right)}_{m}}{{\left(DSIL\right)}_{m}+1}$$

## 3. Numerical Verification

**Figure 4:**
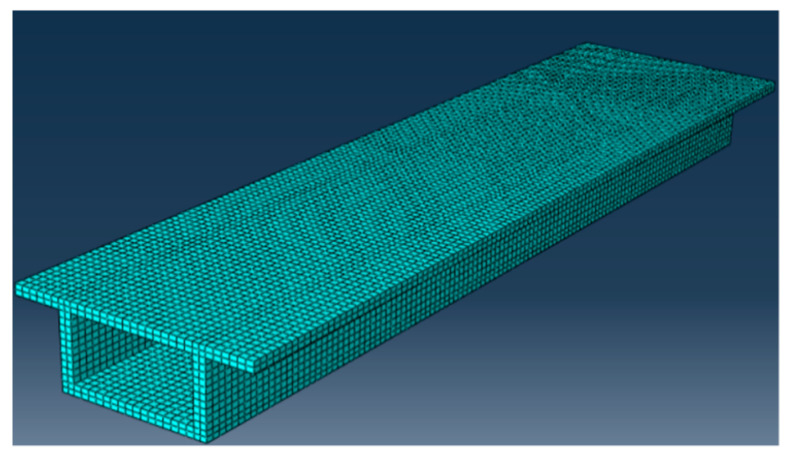
Box girder bridge model.

In order to verify the effectiveness of the above method, a numerical analysis model was established, as shown in Figure 4. The model beam was 18 m in length, 1.6 m in height, 4.68 m in width, and the thickness of the upper and lower plates was 0.2 m. The density of the beam was 2500 kg/m^3^, the elastic modulus was 3.5 × 10^4^ MPa, and the Poisson’s ratio was 0.2. It was divided into 120 elements along the beam length, 15 sensors were arranged at the bottom of the beam, and each sensor was covered with 8 elements. A moving load was applied on the top of the beam, and the strain time history response of each sensor was extracted for analysis.

**Figure 5:**
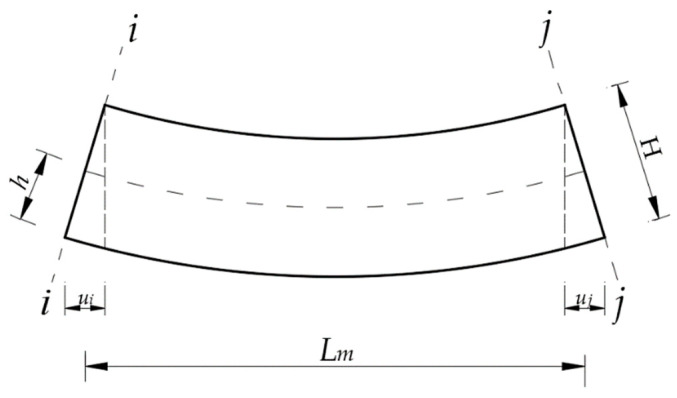
Extraction method of long gauge strain.

Firstly, how to deduce the long gauge strain from the displacement information of nodes was studied. Suppose that a strain sensor with the gauge length of *L_m_* is arranged at the bottom of the beam element (Figure 5), the average strain within the sensor range (i.e., long gauge strain) can be expressed as:

(15) $$\bar{\varepsilon_{m}}=\frac{h}{L_{m}}(v_{i}-v_{j})$$

where *v_i_* and *v_j_* represent the angle displacement of the nodes at both ends of the beam element (section *i–i* and section *j–j*, respectively). In the finite element, the method of extracting the angle displacement is different according to the type of element. The finite element method used in this article cannot extract the rotation angle information directly, and it needs to be converted by Equation (16), where *u_i_* and *u_j_* represent the longitudinal displacement of the element node.

(16) $$\left\{ \begin{array}{l} v_{i}=\frac{u_{i}}{H}\\ v_{j}=\frac{u_{j}}{H} \end{array}\right.$$

**Figure 6:**
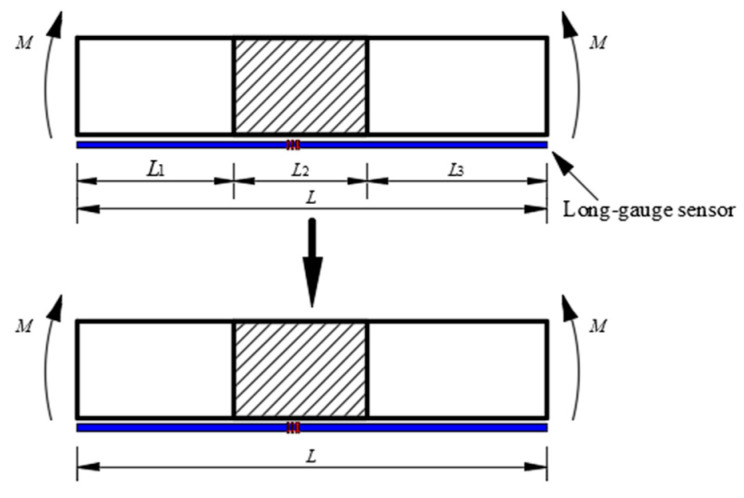
Average stiffness corresponding to the gauge length.

The calculation method of damage extent in the coverage area of long gauge sensors is shown in ref. [25]. Suppose one long-gauge strain sensor with the gauge length *L* is attached on the beam element (Figure 6) and damage occurs in the region of *L*_2_. The average stiffness corresponding to the gauge length using strain equivalent method can be expressed as follows:

(17) $$\frac{ML}{{\left(EI\right)}_{equ}}=\frac{ML_{1}}{EI}+\frac{ML_{2}}{\left(1-\alpha \right)EI}+\frac{ML_{3}}{EI}$$

(18) $${\left(EI\right)}_{equ}=\frac{\left(1-\alpha \right)EIL}{\left(1-\alpha \right)\left(L-L_{2}\right)+L_{2}}$$

where *EI* is the flexural stiffness of the intact beam, *α* is the stiffness reduction coefficient due to the damage, *M* is the bending moment, and ${\left(EI\right)}_{equ}$ represents the equivalent flexural stiffness within the gauge length. Therefore, the average damage extent corresponding to the gauge length can be theoretically calculated by:

(19) $$\bar{\alpha }=\frac{EI-{\left(EI\right)}_{equ}}{EI}=\frac{\alpha L_{2}}{\left(1-\alpha \right)\left(L-L_{2}\right)+L_{2}}$$

A total of 10 working conditions were analyzed: intact condition (C0), single damage condition (C1–C3), two damage condition (C4–C6), and multiple damage condition (C7–C9). The damage setting method was to reduce the unit stiffness of the bottom plate of the box girder in the gauge length section of the sensor, and the specific setting is shown in Table 1. The load type was uniaxial moving load P = 400 kN and the speed was 5 m/s.

### 3.1. Single Damage Case

**Figure 7:**
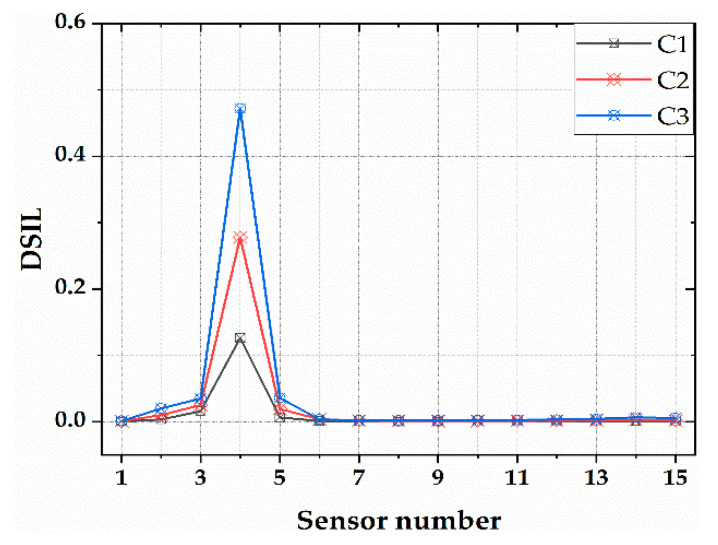
The location identification of single damage.

**Figure 8:**
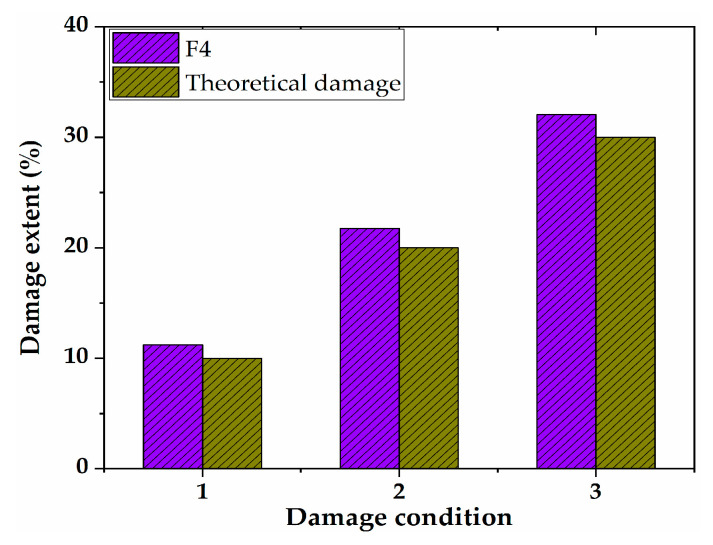
Identification of damage extent.

The proposed damage identification method was used to analyze the working condition C1–C3, and the results are shown in Figure 7. It can be seen from this figure that the DSIL value of the F4 sensor had a large mutation, and the others were basically around zero. The result is consistent with the damage location; therefore, the method can identify the damage location well. In addition, with the increase in the damage degree, the protrusion degree of the DSIL value of the F4 sensor increased gradually, and there was a one-to-one correspondence with the set damage degree, which indicated that the method could not only locate the damage, but also quantify the damage. After inputting the DSIL value into Equation (14), the identification result of the corresponding damage extent could be obtained, as shown in Figure 8. It can be seen that the proposed method can identify the damage extent relatively accurately. The damage extent identified by numerical verification is slightly higher than the theoretical one, and the error is smaller than 3%. 

### 3.2. Multiple Damage Cases

**Figure 9:**
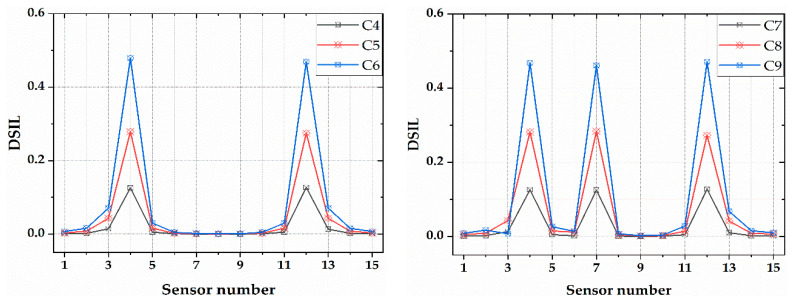
Location identification of two damage and multiple damage.

**Figure 10:**
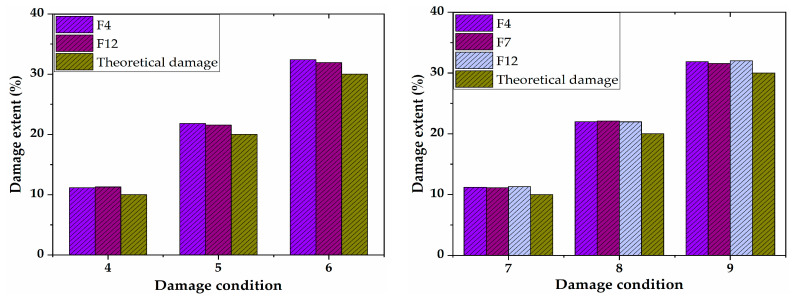
Identification of damage extent of two damage and multiple damage conditions.

Simulation analyses with two or more damage locations were performed to study the damage identification with multiple damage conditions. The simulation results are shown in Figure 9. It can be seen from the figure that the DSIL values of sensors F4 and F12 changed abruptly under two damage conditions, and that the DSIL values of sensors F4, F7 and F12 changed abruptly under multiple damage conditions. The results are consistent with the set damage conditions. The simulation results revealed that the proposed damage identification method is not only suitable for a single damage state, but also suitable for multiple damage states. Similarly, according to Equation (14), the identification results of damage extent could be obtained, which are shown in Figure 10. Similar to the identification result of single damage, the damage extent identified by numerical verification is slightly higher than the theoretical extent.

## 4. Experimental Verification

### 4.1. Experimental Setup

**Figure 11:**
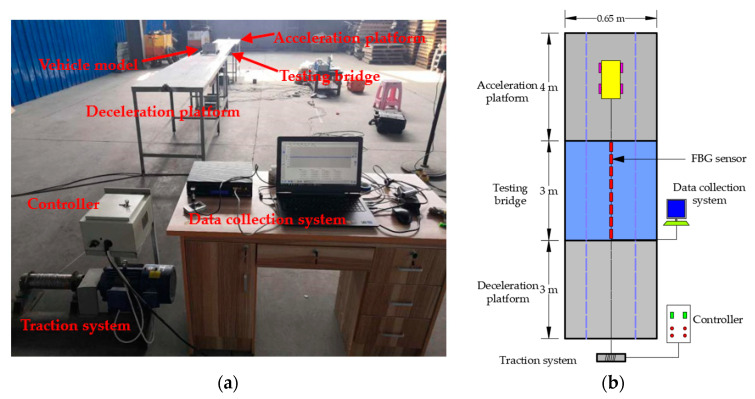
Experimental platform: (**a**) actual scene; (**b**) experiment schematic.

**Figure 12:**
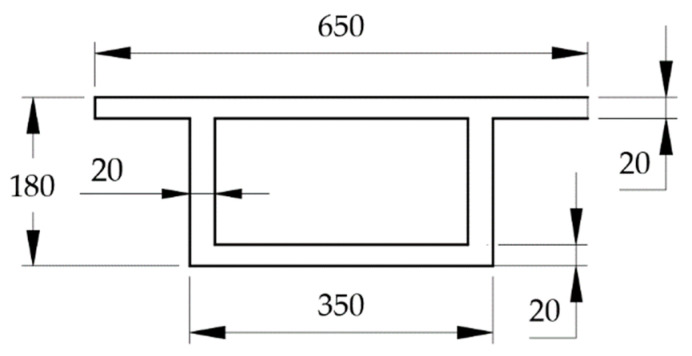
Section size of testing bridge.

To test the feasibility of the proposed method, a series of bridge model experiments were conducted. The experimental platform is shown in Figure 11, including a 4 m acceleration section in front of the bridge, a 3 m test section, and a 3 m deceleration section. The experimental model bridge was made with acrylic material, the performances of which are suitable for simulating concrete bridge structures in the model experiment. Moreover, it is easy to simulate different types of damage with this type of material. The size of the bridge section is shown in Figure 12.

**Figure 13:**
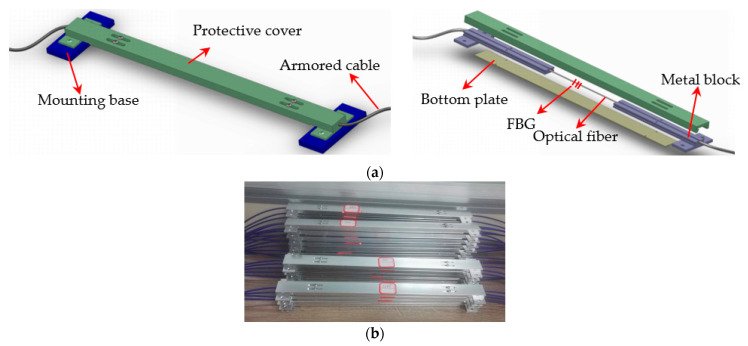
Long-gauge FBG sensor: (**a**) design diagram; (**b**) actual sensors.

In this experiment, eight long-gauge FBG (fiber Bragg grating) strain sensors were symmetrically installed on the bottom of the girder to monitor the strain. As shown in Figure 13, the sensor was composed of fiber grating, two T-shaped metal blocks, an outer protective cover, and armored cable which protected the tail fiber. During the manufacture, the optical fiber needed to be pre-tensioned and encapsulated in the groove between the metal blocks with glue, and the grating was located in the middle of two metal blocks. The measurement sensitivity of the sensor was more than three times higher compared with the ordinary FBG sensor and could be adjusted according to the actual needs. The metal package ensured the durability and service life of the sensor, and it was convenient to be installed and disassembled. The gauge length of the sensor was determined by the distance between the two mounting supports, and the sensors selected in this experiment were 30 cm.

**Figure 14:**
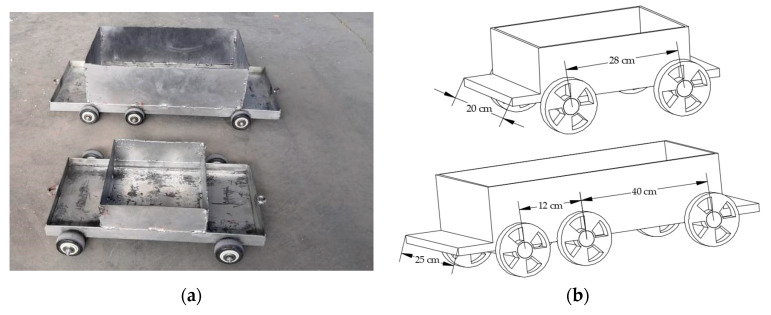
Vehicle models in the experiment: (**a**) actual vehicle models; (**b**) detailed information of the vehicle models.

The experimental vehicle models are shown in Figure 14a, and the vehicle speed was achieved through changing the speed of the motor frequency converter. The vehicle models were divided into two-axle and three-axle vehicles. The specific parameters are shown in Figure 14b. The total weight of the vehicles could be changed with counterweights. The wavelengths of the FBG sensors were acquired with S130 model acquisition instrument of Micron Optics Co., Ltd., and the sampling rate was 500 Hz. In order to study the influence of different parameters on the experimental results, five different vehicle speeds were set in the experiment, and the vehicle counterweights were divided into three levels.

**Figure 15:**
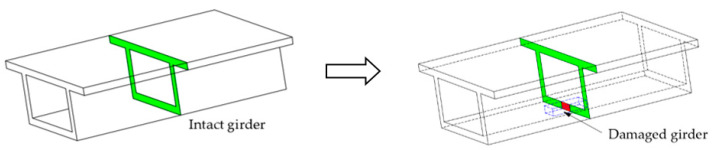
Damage designed method in the experiment.

**Figure 16:**
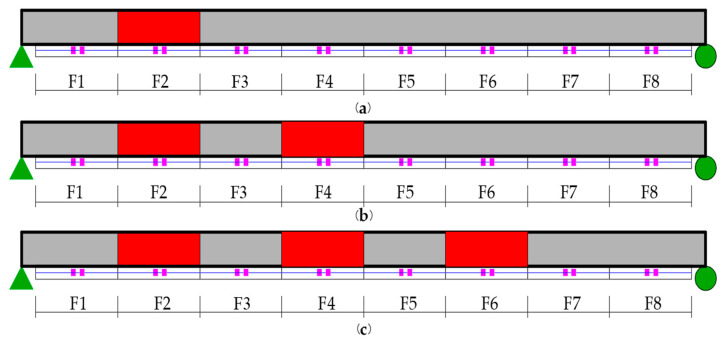
Damage scenarios: (**a**) single damage; (**b**) two damage; (**c**) multiple damage sites.

The damage was realized by cutting the designed length of the box girder at the bottom plate, as shown in Figure 15. The damage extent was calculated through the reduction in the inertia of the section. A total of nine damage conditions were set, including single damage, two damage, and multiple damage scenarios, as shown in Figure 16. The specific design is shown in Table 2, where the parameters include the number and extent of the damage.

### 4.2. Results and Discussion

**Figure 17:**
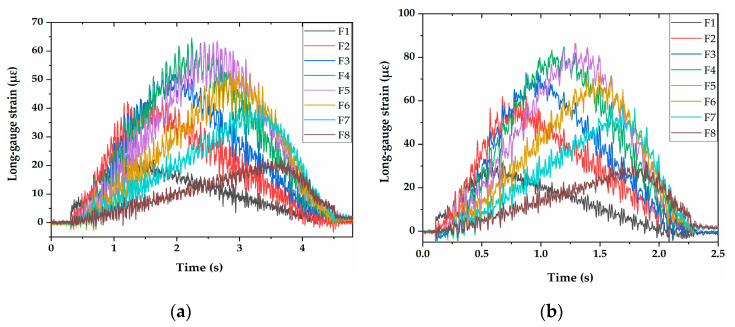
Measured long-gauge strain response of intact testing bridge: (**a**) two-axle vehicle at 0.72 m/s with 10 kg additional weight; (**b**) two-axle vehicle at 1.48 m/s with 10 kg additional weight.

**Figure 18:**
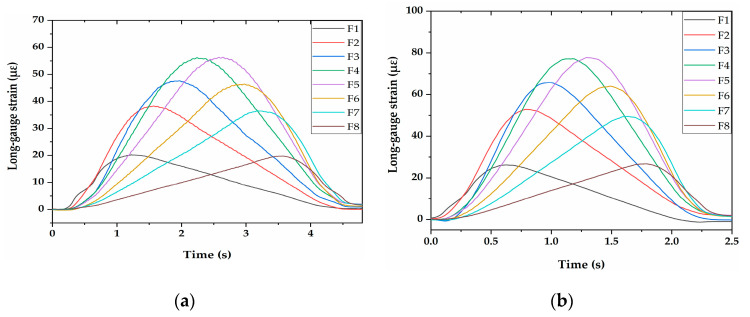
Typical long-gauge strain histories filtered by the EMD method: (**a**) two-axle vehicle at 0.72 m/s with 10 kg additional weight; (**b**) two-axle vehicle at 1.48 m/s with 10 kg additional weight.

The influences of vehicle speed, type and weight on the identified results were studied in the experiments. The measured long-gauge strain response was filtered by the Empirical Mode Decomposition (EMD) method [31], and a typical long-gauge strain history curve was obtained, as shown in Figure 17 and Figure 18. The DSIL values of each sensor under different working conditions can be obtained through inputting the long-gauge strain history curve into the method proposed in this paper. Then, the damage condition of the bridge can be obtained.

#### 4.2.1. Influence of Speed

**Figure 19:**
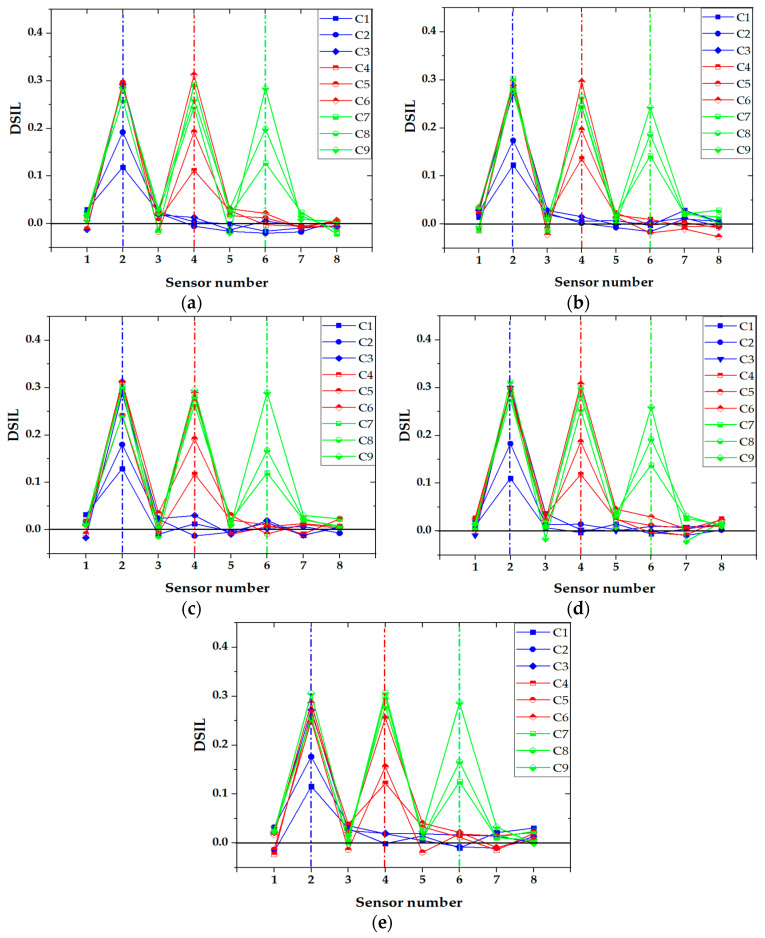
Identification results of damage location: two-axle vehicle with 20 kg additional weight at (**a**) 0.72 m/s; (**b**) 1.11 m/s; (**c**) 1.48 m/s; (**d**) 1.82 m/s; and (**e**) 2.25 m/s.

The vehicle speeds were divided into five types, which were 0.72 m/s, 1.11 m/s, 1.48 m/s, 1.82 m/s, and 2.25 m/s. The damage identification results of two-axle vehicle models with five speed classes are shown in Figure 19. It can be seen that the damage location of the bridge could be well identified under the nine working conditions, and speed had little influence on the identification of damage location. It should be pointed out that the DSIL values obtained with FBG sensors at the boundary and near the damage location fluctuated greatly, which may be related to the dynamic effect. This phenomenon was widespread in the experiment, but did not affect the overall identified effect.

**Figure 20:**
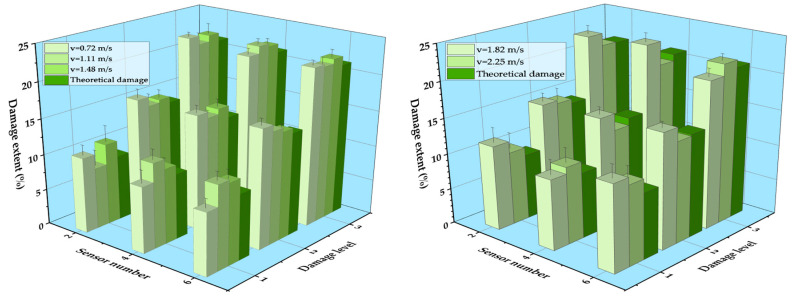
Identification results of damage extent at five different speeds.

According to Equation (14), the damage extent with a relative error could be identified and obtained. The experimental results are shown in Figure 20. It can be seen that the damage extent was accurately identified with the proposed method. The experimental damage extent was generally slightly higher than the theoretical extent, with an average error smaller than 5%, which is within an acceptable range. In addition, it was found that the relative error of the damage identification was large for the small damage extent; it was approximately 2% in damage extent level 1. The relative error decreased with the increase in the damage extent, and its value was smaller than 1% in damage extent level 3. This was due to the reason that the signal-to-noise ratio increased for the specimens with a large damage extent.

#### 4.2.2. Influence of Vehicle Type

**Figure 21:**
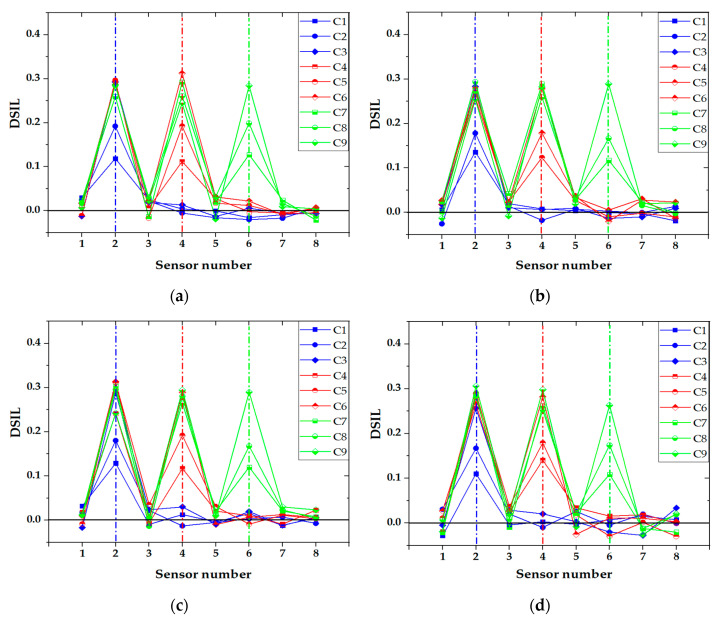
Identification results of damage location: (**a**) two-axle vehicle with a load of 20 kg at 0.72 m/s; (**b**) three-axle vehicle with a load of 20 kg at 0.72 m/s; (**c**) two-axle vehicle with a load of 20 kg at 1.48 m/s; (**d**) three-axle vehicle with a load of 20 kg at 1.48 m/s.

**Figure 22:**
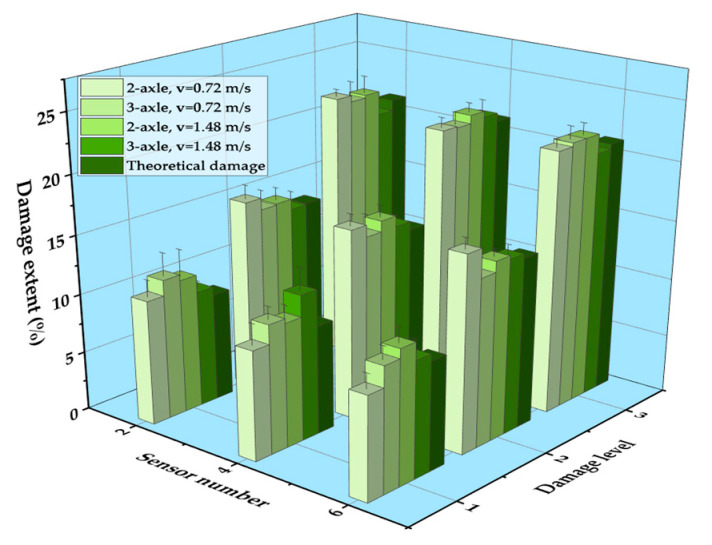
Identification results of damage extent of different vehicle types.

The vehicle types in the experiment were divided into two-axle vehicles and three-axle vehicles. Figure 21 shows the identification results of two-axle vehicles and three-axle vehicles with a 20 kg load at 0.72 m/s and 1.48 m/s, respectively. It can be seen that the location of the damage was accurately identified for both cases, and the vehicle model had little impact on the identification results of damage. Similar with the identification result of speed influence, the fluctuation of DSIL value of the sensor at the boundary and near the damage position still existed, but it did not affect the overall identification effect. Similarly, the identification result of corresponding damage extent with a relative error could be obtained according to the measured DSIL value and Equation (14), as shown in Figure 22. Similar with Figure 20, the measured damage extent was generally higher than the theoretical one, but the identification error was smaller than 5%, which is still acceptable. When the damage extent was small, the relative error was larger. However, the relative error decreased as the damage extent increased. In general, the relative error was smaller than 3% under different vehicle types.

#### 4.2.3. Influence of Vehicle Weight

**Figure 23:**
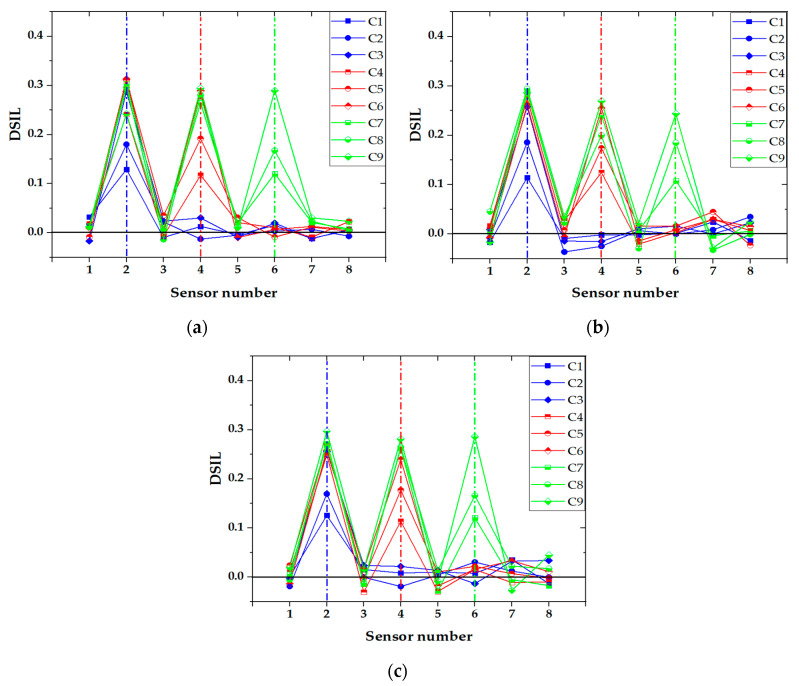
Identification results of damage location: two-axle vehicle at 1.48 m/s with (**a**) 20 kg; (**b**) 30 kg; (**c**) 40 kg additional weight.

**Figure 24:**
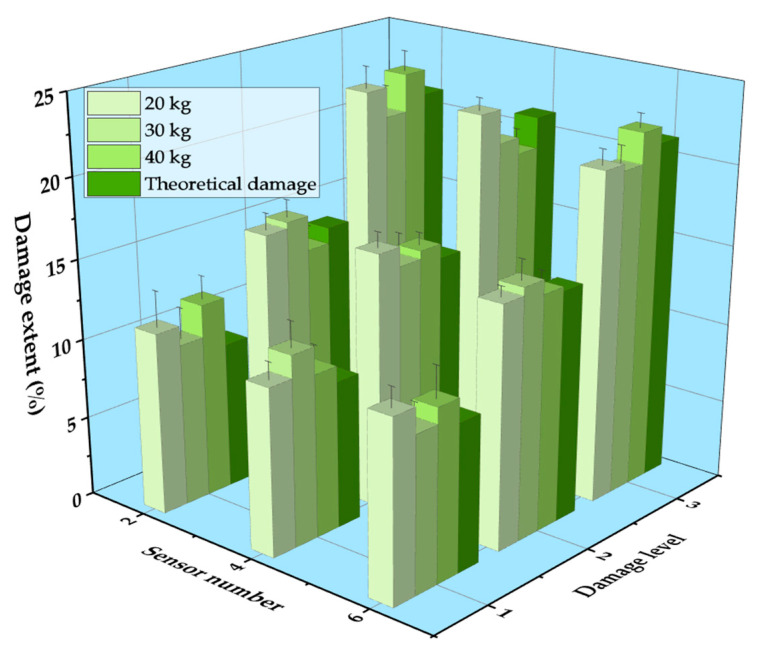
Identification results of damage extent of different vehicle weight.

In the experiment, the additional weight of the vehicle was divided into three grades: 20 kg, 30 kg, and 40 kg. Figure 23 shows the damage identification results of two-axle vehicle at 1.48 m/s with different additional weight conditions. It can be seen that the damage location could be accurately identified under any working conditions, and with the increase in the additional weight on the vehicle, the accuracy of the damage location identification was hardly affected. Consequently, the proposed damage identification method was not affected by the vehicle weight. The identification results of damage extent are shown in Figure 24. The measured damage extent was generally higher than the theoretical extent, and the average identification error of damage extent was smaller than 5%. Moreover, the relative error of damage extent was smaller than 3% under different vehicle weight conditions.

## 5. Conclusions

A novel damage identification method for box girder bridge structures was proposed through the relationship between strain difference index DSIL and damage extent. In addition, a series of numerical simulation and experiments were conducted to verify the proposed method. The main conclusions are as follows:

1. The regularization index DSIL before and after the damage of bridge structures is related to the stiffness of the bridge and the installation position of the sensor, which can be used to identify the damage. When the damage occurs in the gauge length of the sensor, the value of DSIL deviates from zero, and the damage location is, thus, identified.
2. Through the verification of numerical simulation, the method can locate the damage accurately, and has a certain ability to identify the damage extent.
3. Through a series of experiments, the influence of speed, vehicle type, and vehicle weight on the identification method were studied. The experimental results show that the method is not affected by these parameters and the damage location can be accurately identified under various working conditions.
4. When the damage was analyzed quantitatively, it was found that the measured damage extents were generally higher than the theoretical one, and the average identification error of damage extent was smaller than 5%. Moreover, the relative error decreased as the damage extent increased. In general, the relative error was smaller than 3% under different working conditions.

## Figures and Tables

**Table 1:** Damage condition design.

Condition	Damage Location	Damage Extent	Damage Location	Damage Extent	Damage Location	Damage Extent
C0	F4		F7		F12	
C1	F4	10%	F7		F12	
C2	F4	20%	F7		F12	
C3	F4	30%	F7		F12	
C4	F4	10%	F7		F12	10%
C5	F4	20%	F7		F12	20%
C6	F4	30%	F7		F12	30%
C7	F4	10%	F7	10%	F12	10%
C8	F4	20%	F7	20%	F12	20%
C9	F4	30%	F7	30%	F12	30%

**Table 2:** Damage conditions in the experiment.

Condition	Damage Location	Damage Extent	Damage Location	Damage Extent	Damage Location	Damage Extent
C0	F2		F4		F6	
C1	F2	9.19%	F4		F6	
C2	F2	14.43%	F4		F6	
C3	F2	21.25%	F4		F6	
C4	F2	21.25%	F4	9.19%	F6	
C5	F2	21.25%	F4	14.43%	F6	
C6	F2	21.25%	F4	21.25%	F6	
C7	F2	21.25%	F4	21.25%	F6	9.19%
C8	F2	21.25%	F4	21.25%	F6	14.43%
C9	F2	21.25%	F4	21.25%	F6	21.25%

## Data Availability

Not applicable.

## References

[1] Cardini A.J., DeWolf J.T. (2009). Long-term structural health monitoring of a multi-girder steel composite bridge using strain data. Struct. Health Monit..

[2] Adewuyi A.P., Wu Z.S. (2009). Assessment of vibration-based damage identification methods using displacement and distributed strain measurements. Struct. Health Monit..

[3] Pang L., Liu J., Harkin J. (2020). Case Study-Spiking Neural Network Hardware System for Structural Health Monitoring. Sensors.

[4] Maheshwari M., Tjin S.C., Ching W.W., Asundi A. (2015). FBG and FOPS for local and global structural health monitoring on a single fiber. Smart Mater. Struct..

[5] Kim B., Min C., Kim H., Cho S., Oh J., Ha S.-H., Yi J.-H. (2019). Structural Health Monitoring with Sensor Data and Cosine Similarity for Multi-Damages. Sensors.

[6] Catbas F.N., Aktan A.E. (2002). Condition and damage assessment: Issues and some promising indices. J. Struct. Eng..

[7] Shih H.W., Thambiratnam D.P., Chan T.H.T. (2013). Damage detection in slab-on-girder bridges using vibration characteristics. Struct. Control Health Monit..

[8] Koh B.H., Dyke S.J. (2007). Structural health monitoring for flexible bridge structures using correlation and sensitivity of modal data. Comput. Struct..

[9] Zhang J., Guo S.L., Wu Z.S., Zhang Q.Q. (2015). Structural identification and damage detection through long-gauge strain measurements. Eng. Struct..

[10] Roveri N., Carcaterra A. (2012). Damage detection in structures under traveling loads by Hilbert-Huang transform. Mech. Syst. Signal Process..

[11] Nayek R., Mukhopadhyay S., Narasimhan S. (2018). Mass normalized mode shape identification of bridge structures using a single actuator-sensor pair. Struct. Control Health Monit..

[12] Glisic B., Inaudi D. (2012). Development of method for in-service crack detection based on distributed fiber optic sensors. Struct. Health Monit..

[13] Yao Y., Tung S.T.E., Glisic B. (2014). Crack detection and characterization techniques–an overview. Struct. Control Health Monit..

[14] He W.Y., Ren W.X., Zhu S.Y. (2017). Damage detection of beam structures using quasi-static moving load induced displacement response. Eng. Struct..

[15] Doebling S.W., Farrar C.R., Prime M.B. (1998). A summary review of vibration-based damage identification methods. Shock Vib. Dig..

[16] Lee J., Kim S. (2007). Structural damage detection in the frequency domain using neural networks. J. Intell. Mater. Syst. Struct..

[17] Ntotsios E., Papadimitriou C., Panetsos P. (2009). Bridge health monitoring system based on vibration measurements. Bull. Earthq. Eng..

[18] Li H.L., Lu Z.R., Liu J.K. (2016). Identification of distributed damage in bridges from vehicle-induced dynamic responses. Adv. Struct. Eng..

[19] Liu C., Gong Y., Laflamme S., Phares B., Sarkar S. (2016). Bridge damage detection using spatiotemporal patterns extracted from dense sensor network. Meas. Sci. Technol..

[20] Nguyen K.D., Chan T.H., Thambiratnam D.P. (2016). Structural damage identification based on change in geometric modal strain energy-eigenvalue ratio. Smart Mater. Struct..

[21] Li S.Z., Wu Z.S. (2007). Development of distributed long-gauge fiber optic sensing system for structural health monitoring. Struct. Health Monit..

[22] Liu Y., Zhang S. (2018). Damage Localization of Beam Bridges Using Quasi-Static Strain Influence Lines Based on the BOTDA Technique. Sensors.

[23] Sun L.M., Zhang W., Nagarajaiah S. (2019). Bridge real-time damage identification method using inclination and strain measurements in the presence of temperature variation. J. Bridge Eng..

[24] Xu Z.D., Li S., Zeng X. (2018). Distributed strain damage identification technique for long-span bridges under ambient excitation. Int. J. Struct. Stab. Dyn..

[25] Hong W., Wu Z., Yang C. (2012). Investigation on the damage identification of bridges using distributed long-gauge dynamic macrostrain response under ambient excitation. J. Intell. Mater. Syst. Struct..

[26] Hong W., Cao Y., Wu Z. (2016). Strain-Based Damage-Assessment Method for Bridges under Moving Vehicular Loads Using Long-Gauge Strain Sensing. J. Bridge Eng..

[27] Chen S.Z., Feng D.C., Han W.S. (2020). Comparative study of damage detection methods based on long-gauge FBG for highway bridges. Sensors.

[28] Wu B., Wu G., Lu H., Feng D.C. (2017). Stiffness monitoring and damage assessment of bridges under moving vehicular loads using spatially-distributed optical fiber sensors. Smart Mater. Struct..

[29] Ojio T., Yamada K. Bridge Weigh-in-Motion Systems Using Stringers of Plate Girder Bridges. Proceedings of the Third International Conference on Weigh-in-Motion (ICWIM3).

[30] Guo J.Q. (2008). Design Theory of Box Girder.

[31] Peng Z.K., Peter W.T., Chu F.L. (2005). An improved Hilbert–Huang transform and its application in vibration signal analysis. J. Sound Vib..

